# Oxidative Stress and Modification of Renal Vascular Permeability Are Associated with Acute Kidney Injury during *P. berghei* ANKA Infection

**DOI:** 10.1371/journal.pone.0044004

**Published:** 2012-08-31

**Authors:** Rosa Maria Elias, Matheus Correa-Costa, Claudiene Rodrigues Barreto, Reinaldo Correia Silva, Caroline Y. Hayashida, Ângela Castoldi, Giselle Martins Gonçalves, Tarcio Teodoro Braga, Renato Barboza, Francisco José Rios, Alexandre Castro Keller, Marcos Antonio Cenedeze, Meire Ioshie Hyane, Maria Regina D'Império-Lima, Antônio Martins Figueiredo-Neto, Marlene Antônia Reis, Cláudio Romero Farias Marinho, Alvaro Pacheco-Silva, Niels Olsen Saraiva Câmara

**Affiliations:** 1 Disciplina de Nefrologia, Departamento de Medicina, Universidade Federal de São Paulo, São Paulo, Brazil; 2 Laboratório de Imunobiologia de Transplantes, Departamento de Imunologia, Universidade de São Paulo, São Paulo, Brazil; 3 Departamento de Imunologia, Universidade Federal de São Paulo, São Paulo, Brazil; 4 Instituto de Física, Universidade de São Paulo, São Paulo, Brazil; 5 Divisão de Patologia, Universidade Federal do Triângulo Mineiro, Uberaba, Brazil; 6 Departamento de Parasitologia, Instituto de Ciências Biomédicas, Universidade de São Paulo, São Paulo, Brazil; 7 Instituto Israelita de Ensino e Pesquisa Albert Einstein, São Paulo, Brazil; State University of Campinas, Brazil

## Abstract

Malaria associated-acute kidney injury (AKI) is associated with 45% of mortality in adult patients hospitalized with severe form of the disease. However, the causes that lead to a framework of malaria-associated AKI are still poorly characterized. Some clinical studies speculate that oxidative stress products, a characteristic of *Plasmodium* infection, as well as proinflammatory response induced by the parasite are involved in its pathophysiology. Therefore, we aimed to investigate the development of malaria-associated AKI during infection by *P. berghei* ANKA, with special attention to the role played by the inflammatory response and the involvement of oxidative stress. For that, we took advantage of an experimental model of severe malaria that showed significant changes in the renal pathophysiology to investigate the role of malaria infection in the renal microvascular permeability and tissue injury. Therefore, BALB/c mice were infected with *P. berghei* ANKA. To assess renal function, creatinine, blood urea nitrogen, and ratio of proteinuria and creatininuria were evaluated. The products of oxidative stress, as well as cytokine profile were quantified in plasma and renal tissue. The change of renal microvascular permeability, tissue hypoxia and cellular apoptosis were also evaluated. Parasite infection resulted in renal dysfunction. Furthermore, we observed increased expression of adhesion molecule, proinflammatory cytokines and products of oxidative stress, associated with a decrease mRNA expression of HO-1 in kidney tissue of infected mice. The measurement of lipoprotein oxidizability also showed a significant increase in plasma of infected animals. Together, our findings support the idea that products of oxidative stress, as well as the immune response against the parasite are crucial to changes in kidney architecture and microvascular endothelial permeability of BALB/c mice infected with *P. berghei* ANKA.

## Introduction

Every year, there are about 800 thousand people dying from severe form of malaria (World Malaria Report). Malaria-associated acute kidney injury (AKI), one of the three major life-threatening well-know causes of death in *P. falciparum* [1], [2] and *P. vivax* severe malaria [3], [4], occurs between 1–4% of hospitalized adult [5] with a mortality that can reach up to 45% [1]. The pathogenesis of malaria-associated AKI is multifactorial and not well characterized, but several hypotheses suggest involvement of cytoadherence of iRBC, proinflammatory response as well as nephrotoxicity due to oxidative stress. It is well-establish that pathogenesis of severe malaria is associated with an up regulation of proinflammatory cytokines [6], [7]. During intra-erythrocytic phase, the consumption of hemoglobin by parasites gives rise of considerable amounts of free heme (Fe^+3^), a molecule that have the ability to induce oxidative stress [8]. The oxidative stress mediated by free heme has been implicated in lipoprotein oxidation [9] and serious damage in different organs such as the kidneys [10] through generation of reactive oxygen intermediates, and nitrogen intermediates (ROI and NO) by host cells. Moreover, ox-LDL upregulates the expression of adhesion molecules, facilitating the cytoadherence of infected erythrocytes [11]. The sequestration and adhesion of infected red blood cells (iRBC) to endothelial cells compromises vascular permeability of vital organs [12]. The changes in the endothelial permeability contribute to alterations of microvascular pattern and proinflammatory cytokine release [13], [14]. According to this, the main focus of this study was to evaluate how P. berghei ANKA infection affects or modifies kidney pathophysiology leading to cell injury, as well as the involvement of oxidative stress that occurs during plasmodium infection, determine the influence of renal endothelial modifications to development of malaria-associated AKI and also characterize how HO-1 may participate in both protection and pathogenesis of clinical outcome.

**Figure 1:**
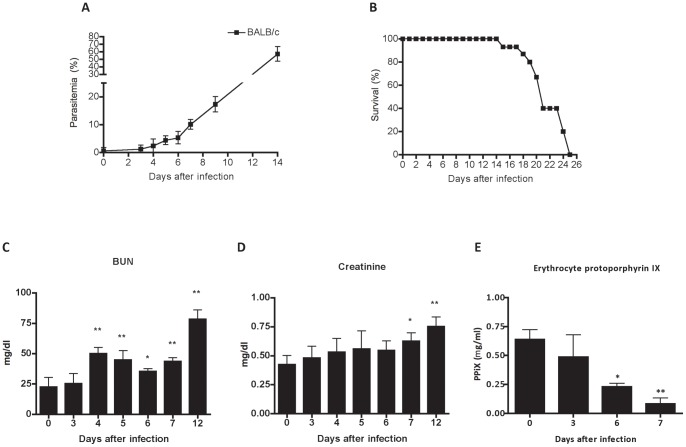
Impairment of renal function during *P. berghei* ANKA malaria infection. (A) Parasitemia, and (B) survival of BALB/c mice infected with 10^6^ parasitized erythrocytes by *P. berghei* ANKA. Renal function was assessed by (C) plasma creatinine, (D) blood urea nitrogen (BUN) and (E) quantification of erythrocyte protoporphyrin estimated on different days after infection. Results represent the mean of 5–10 animals per group ± standard deviation. One-way ANOVA with Bonferroni post-test was performed to renal assessment using GraphPad Prism. * P<0.05 vs the control group – day 0, ** p<0.01 vs the control group – 0 days.

## Materials and Methods

### Mice

BALB/c mice were bred and housed in specific pathogen-free facilities of the Instituto de Ciências Biomédica IV (USP) and CEDEME (UNIFESP – Escola Paulista de Medicina, EPM). The Animal Care Committee of the UNIFESP approved all protocols.

**Figure 2:**
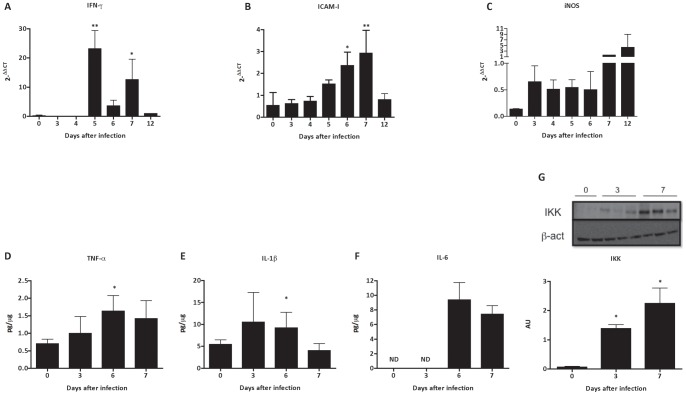
Effect of *P. berghei* ANKA malaria infection in renal pro-inflammatory response. mRNA expression of (A) IFN-γ, (B) ICAM-1 and (C) iNOS in renal tissue of BALB/c mice infected with 10^6^ parasitized erythrocytes by *P. berghei* ANKA. Renal tissue protein expression of (D) TNF-α, (E) IL-1β and (F) IL-6. (G) Representative image and graphic quantification of bands expressed of an IKK western blot. The graphs of A to F represent the average of 3–5 animals per group ± standard deviation. One-way ANOVA with Bonferroni post-test was performed to mRNA expression and quantification of IKK using GraphPad Prism. Unpaired Student-t test was performed to renal tissue protein expression using GraphPad Prism. * P<0.05 vs the control group – day 0, ** p<0.01 vs the control group – 0 days.

### Parasites, infection and disease assessment

BALB/c mice were infected by intraperitoneal (i.p.) inoculation of 10^5^ red blood cells infected with green fluorescent protein (GFP)-transgenic *P. berghei* ANKA, clone 259Cl2 [15], [16]. Daily parasitemia were determined by flow cytometry from day 3 after inoculation. Blood, urine and kidney samples were collected at different days after infection. Uninfected mice were used as control group.

**Figure 3:**
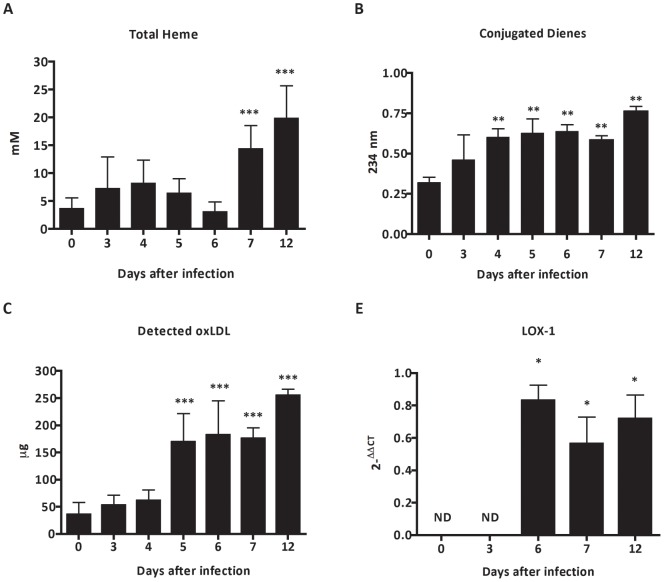
Assessment of products of oxidative stress during malaria-associated AKI. (A) Plasmatic quantification of toxic heme (B), detection of conjugated dienes by Cooper and (C) quantification of plasma levels of oxidized low density lipoprotein in BALB/c mice infected with 10^6^ parasitized erythrocytes by *P. berghei* ANKA. (D) mRNA expression of LOX-1 in renal tissue during infection in BALB/c mice. The results represent the average of 5–10 animals per group ± standard deviation. One-way ANOVA with Bonferroni post-test was performed using GraphPad Prism. * P<0.05 vs the control group – day 0, ** p<0.01 vs the control group – 0 days, *** p <0.001 vs the control group – 0 days.

### Renal vascular permeability

The renal microvascular modification was assessed by extravasations of Evans blue dye from the kidneys parenchyma as previously described [17]. Evans blue dye concentration was measured through absorbance at 620 nm. Data was presented as microgram of Evans blue dye per gram of tissue.

### Assessment of renal function

Blood urea nitrogen (BUN) was measured using a Labtest Kit (Labtest, Minas Gerais, Brazil) and serum creatinine was measured by Jaffé's modified method. Urinary protein/creatinine ratios were analyzed by using a Labtest kit (Labtest, Minas Gerais, Brazil).

**Figure 4:**
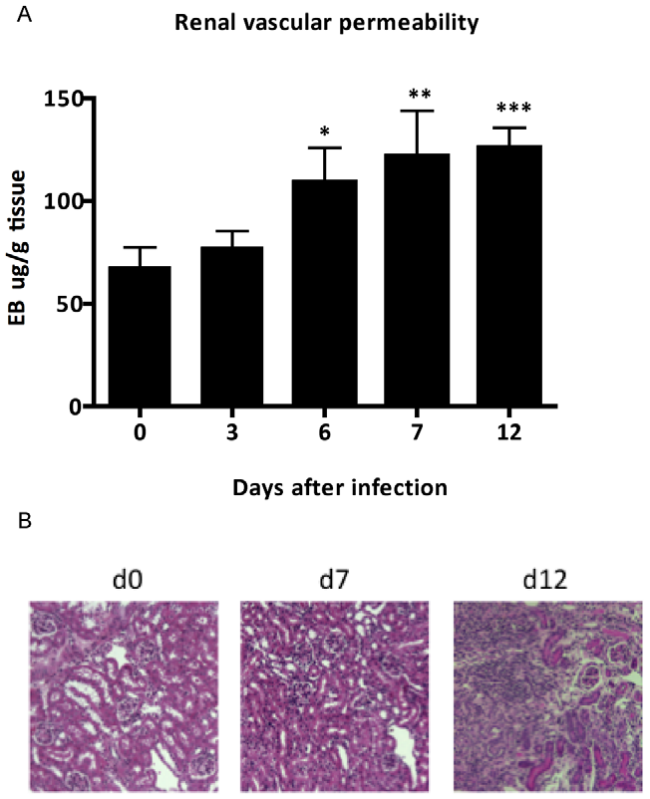
*P. berghei* ANKA malaria infection induces endothelium injury and changes in renal architecture. (A) Renal microvascular permeability change assessed by Evans blue dye and (B) representative pathophysiology of renal tissue stained with hematoxylin-eosin (HE) and examined in light microscopy (Leica DM LB2, Leica Microsystems). Each graph represents the mean of 5–10 animals per group ± standard deviation. One-way ANOVA with Bonferroni post-test was performed using GraphPad Prism. * P<0.05 vs. the control group – day 0, ** p<0.01 vs. the control group – day 0, *** p<0.001 vs. the control group – day 0.

**Figure 5:**
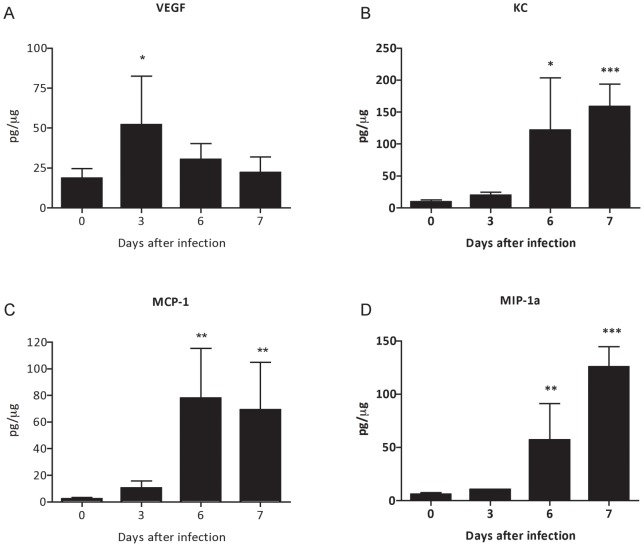
Monocytes, neutrophils and polymorphonuclear leukocytes are recruited to renal tissue in *P. berghei* ANKA malaria infection. Renal tissue protein expression of (A) VEGF, (B) KC, (C) MCP-1 and (D) MIP-1α during *P. berghei* ANKA infection. Each graph represents the mean of 5–10 animals per group ± standard deviation. Unpaired Student-t test was performed using GraphPad Prism. * P<0.05 vs. the control group - day 0, ** p<0.01 vs. the control group - day 0, *** p<0.001 vs. the control group – day 0.

### Total heme determination

Total heme determination in plasma was quantified using a colorimetric assay according to the manufacturer's instructions (QuantiChrom heme assay kit, Bioassay Systems) as previously described [18].

**Figure 6:**
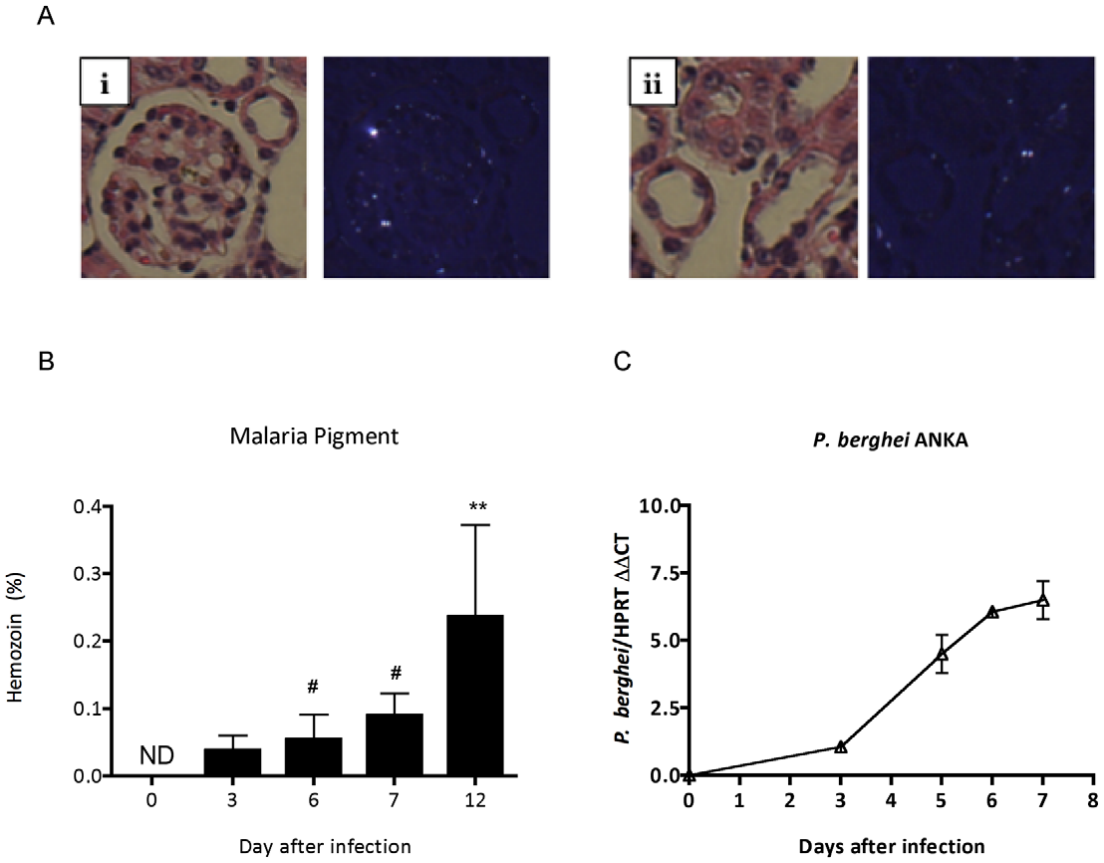
Analysis of sequestration of infected red blood cell in renal tissue. (A) Representative figure of presence of malaria pigment hemozoin in renal tissue sections visualized by hematoxylin and eosin, or under polarized light. Detection of hemozoin at glomeruli (**i**) and vascular endothelium (**ii**). (B) Hemozoin quantification in histological section of renal tissue. (C) *P. berghei* ANKA mRNA quantified by qPCR in renal tissue of BALB/c infected mice. Each graph represents the mean of 5–10 animals per group ± standard deviation. One-way ANOVA with Bonferroni post-test was performed using GraphPad Prism. *** p<0.001 vs. the control group – day 0, # p<0.01 vs. day 12.

**Figure 7:**
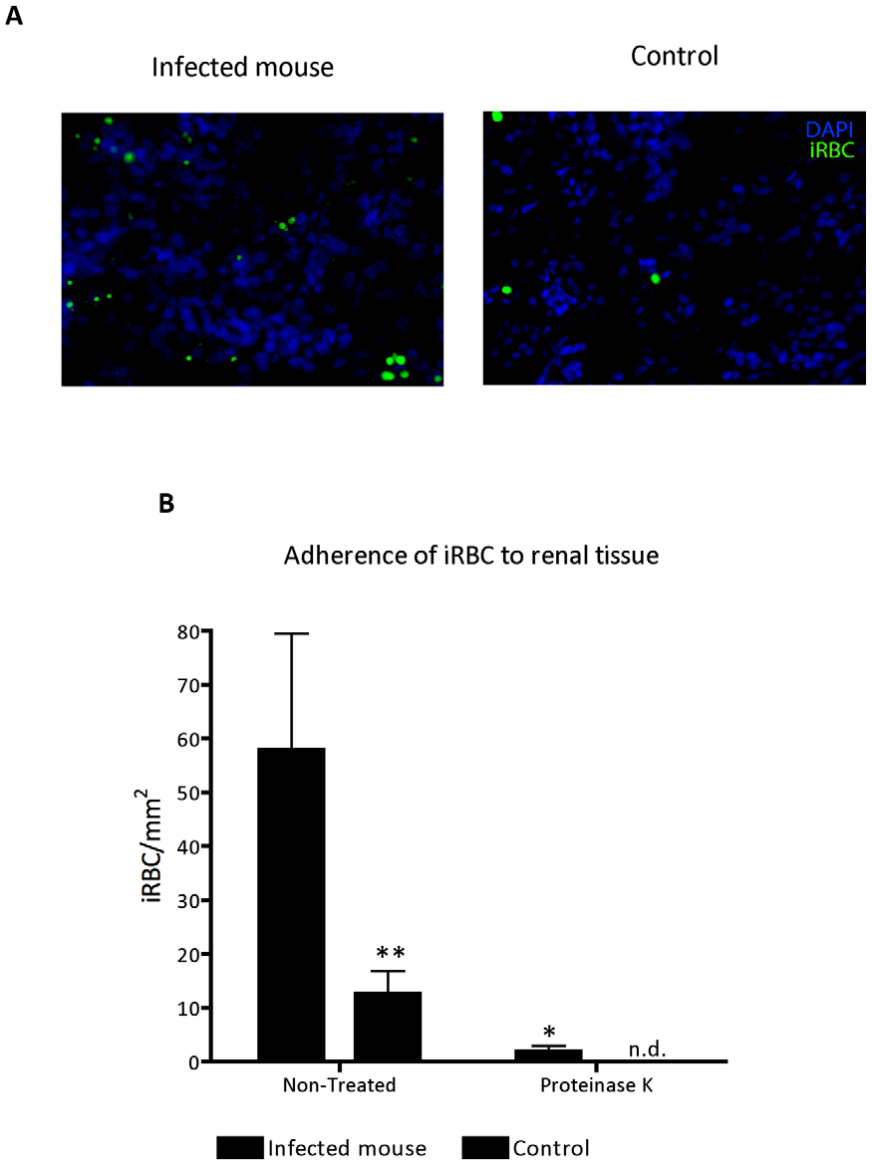
*Ex vivo* adherence of *P. berghei* ANKA^GFP^ iRBC to renal tissue. (**A**) Representative microscopic image of the *ex vivo* adherence assays showing iRBC adhering to renal tissue sections from control (right) and infected (left) mice (200X magnification). (**B**) Adhesion of iRBC treated or not with proteinase K prior incubation with the frozen kidney sections. All data represent the number of bound iRBC per area. (mean±s.e.m). Two-way ANOVA with Bonferroni post-test was performed using GraphPad Prism. *P<0.05; **P<0.01. (n.d.: not-detected).

### Porphyrin extraction

The assessment of erythrocytes Protoporphyrin IX (PpIX) fluorescence was evaluated as previously described [19]. To spectrofluorometric determination of PpIX in samples, a standard curve of commercial metal-free PpIX (Sigma Chemical Company, St. Louis, Mo., USA) 0.01 mg/mL to 2 mg/mL was constructed. The samples and standard curve was exciting in 405 nm and the absorbance was measured between 610 nm and 730 nm by Synergy Mx (BioTek Instruments, Inc). A standard curve of PpIX was used to convert absorbance into concentrations of PpIX. Data was presented as microgram of mg/ml.

**Figure 8:**
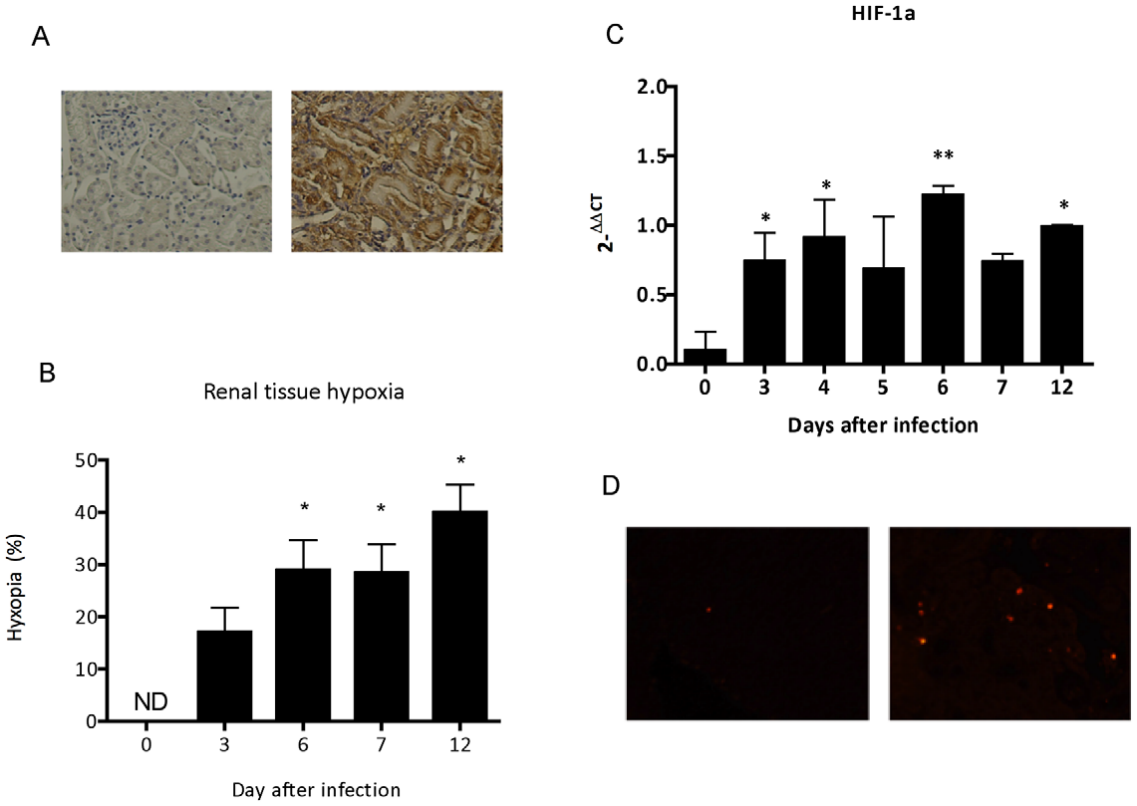
Evaluation of hypoxia, HIF-1α in renal tissue and apoptosis during malaria-associated AKI. (A) Representative immunohistochemistry, and (B) quantification of renal hypoxia in control and *P. berghei* ANKA infected mice. (C) mRNA expression of HIF-1α in renal tissue. (D) Evaluation of apoptosis in kidney section of control and *P. berghei* ANKA infected mice. Each graph represents the mean of 5–10 animals per group ± standard deviation. One-way ANOVA with Bonferroni post-test was performed using GraphPad Prism. * P<0.05 vs control group – day 0, ** p<0.01 vs control group – day 0.

### Determination of indirect bilirubin

Indirect bilirubin was measured in plasma sample using a commercial kit (Labtest, Lagoa Santa, MG, Brazil) following the manufacturer's protocol. Samples were read at 540 nm, and the results were expressed as mg of indirect bilirubin per dl.

**Figure 9:**
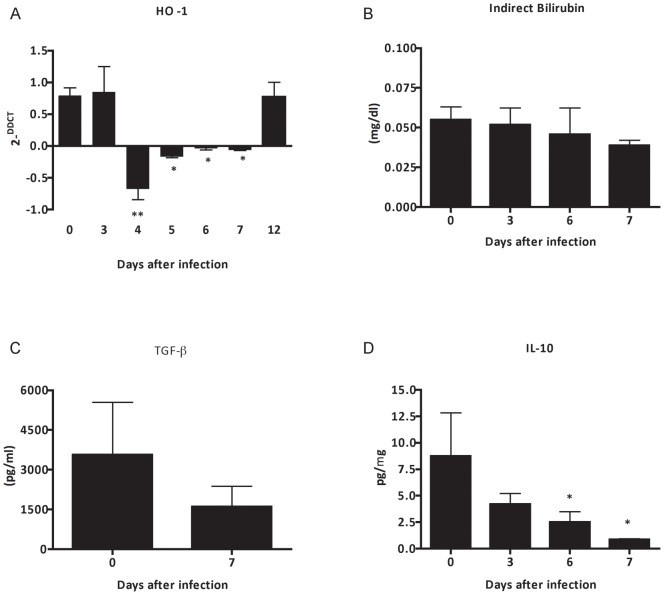
Lack of cytoprotective defense mechanisms during *P. berghei* ANKA malaria infection. (A) mRNA expression of HO-1, (B) plasma level of indirect billirubin and (C) TGF-β and (D) renal tissue protein expression of IL-10 from BALB/c mice infected with 10^6^ parasitized erythrocytes by *P. berghei* ANKA. The graphs represent the average of 3–5 animals per group ± standard deviation. One-way ANOVA with Bonferroni post-test was performed to mRNA expression of HO-1 and plasma level of indirect billirubin using GraphPad Prism. Unpaired Student-t test was performed to renal tissue protein expression using GraphPad Prism. * P<0.05 vs the control group – day 0.

### Bioplex

Kidney tissues were lysed in RIPA buffer with protease inhibitor. A BioPlex mice Plex cytokine assay kit (BioRad Laboratories, Inc., Hercules, CA, USA) was used to test samples for the presence of 15 molecules. The assay was read on the BioPlex suspension array system, and the data were analyzed using BioPlex Manager software version 4.0. Standard curves ranged from 32,000 to 1.95 pg/mL.

### Measurement of TGF-β

Total TGF-β was measured in the plasma using ELISA assay, according to the manufacturer's instructions (TGFβ1 EmaxH, Promega, Madison, USA) previously used [20]. Results are expressed as pg/mg of TGF-β protein.

### Detection of hypoxia tissue

Pimonidazole (Chemicon International, Inc., CA, USA) was injected (i.p.) 1h at a dose of 60mg/kg body weight before euthanasia and hypoxic regions of the renal tissue was detected by the Hypoxyprobe-1 Pab2627 (1∶500) primary antibody as described previously [21]. Immunohistochemistry with cleaved caspase-3 antibody (diluted 1∶1000 (Asp175), Cell Signaling Technology, Beverly, MA, USA) was also performed as previously described [17]. The presence of pimonidazole-HCL in renal tissue was calculated as a percentage in the cortex and medulla using a computer program for image analysis (KS300, Zeiss system). The average area of each sample was calculated in each kidney.

### RNA extraction and Real Time PCR quantification to gene expression

Total RNA was isolated from kidney tissue using TRIzol reagent (Invitrogen, USA). First-strand complementary DNAs (cDNAs) were synthesized using a MML-V reverse transcriptase kit (Promega, USA). Real Time PCR (qPCR) was performed using the TaqMan real-time PCR assay (Applied Biosystem, USA). mRNA expression was normalized to HPRT and the values are expressed relative to a reference sample (the calibrator). The Ct (threshold cycle) for the target gene and for the internal control was determined for each sample. A triplicate of each sample was done. The relative expression of mRNA expression was calculated by 2-ΔΔCT. All the experimental samples were expressed as n-fold difference relative to the calibrator.

### Measurement of lipoprotein oxidizability

Oxidized low-density lipoprotein (ox-LDL) in plasma was measured by ELISA kit (ox-LDL β2GP, Cayman, USA) following the manufacturer's protocol. Results were calculated against a standard curve and expressed in serum ox-LDL (μg). The rate of formation of conjugated dienes was measured at plasma as previously described [22]. Briefly, whole plasma was diluted 150-fold and the absorption of conjugated dienes performed by spectrophotometer in a quartz plate at 37°C and measured at 234 nm.

### Kidney histology analysis

To observe changes in the pathophysiology of the kidney associated with severe malaria, kidney was fixed in 10% formalin for further processing. Paraffin-embedded kidney sections was stained with hematoxylin-eosin (HE) and examined in light microscopy (Leica DM LB2, Leica Microsystems). Renal morphometric analysis was performed in a blinded manner by a single observer. The presence of acute tubular necrosis (ATN) was estimated in 4-lm-thick hematoxylin and eosin-stained sections. All microscopic fields of each slide were examined at final magnification of 250x. Tubular injury was evaluated using a semi quantitative scale, on which the percentage of cortical and outer medulla tubules showing epithelial necrosis was assigned a score as follows: 0 for 10%, 1 for 10–25%, 2 for 26–75%, and 3 for 75%. The mean of all area analyzed was plotted and compared between groups.

### Malaria pigment detection in kidney sections

The presence of hemozoin was assessed at structures such as the glomeruli, subcapsular cortex and large vessels observed under polarized light. Photos of at least 5 different fields in each slide were taken and the pictures digitalized at HP Scanjet 2400.

### Synchronization and enrichment of parasitized erythrocytes

Red blood cells were collected from infected animals with 30% parasitemia by cardiac puncture and placed in culture medium RPMI 1640 (Gibco) supplemented with 5% of fetal bovine serum (FBS). To obtain mature forms (trophozoites/schizonts), *P. berghei* ANKA^GFP^ infected red blood cells (iRBC) were synchronized as describe previously [23]. Briefly, the parasites were maintained *in vitro* at 37°C for 18h in atmosphere containing 5% of CO2, 85% of N_2_ and 10% of O_2_. The erythrocytes were then enriched by magnetic separation column (MACS BEADS, Miltenyi Biotec, USA), resulting in cell populations with approximately 95% of iRBC.

### Cytoadherence assays

To verify the capacity of erythrocytes infected with *P. berghei* ANKA^GFP^ to adhere to renal tissue, synchronized iRBC were overlaid on frozen renal sections for 1h at 37^o^C. Fifty microliters of synchronized iRBC suspension, at the concentration of 10^8^/ml, were overlaid on frozen renal sections for 60 minutes at 37°C in a humid chamber. After washing the unbound cells, the slides were mounted with Vectashield contained DAPI (Vector Lab. Bruglingame, CA, USA) and examined under fluorescence microscopy (magnification 200X). The number of iRBC adhering to kidney sections was determined in a blind fashion, counting 10 fields in each section. As negative control, synchronized iRBC were pre-treated with 40 µg/ml Proteinase K (Sigma) for 30 minutes at 37^o^C. After washing, iRBC were overlaid on renal sections as described above.

### Assessment of apoptosis

To detect apoptotic cells, the *In situ* Cell Death Detection Kit TMR red (Roche Diagnostics GmbH, Mannheim, Germany) was used (TUNEL technology).

### Western blotting analysis

Primary mouse IKKα antibody (SC-166231, Santa Cruz Biotechnology, Inc) was used following manufacturer-recommended dilutions, followed by a peroxidase-conjugated anti-mouse IgG antibody (Jackson ImmunoResearch Laboratories, WestGrove, USA). Mouse primary anti–β-tubulin or anti-β-actin antibody (Sigma, St. Louis, USA) was also used to confirm and estimate the loading and the transfer. We used the software GeneSnap (Syngene, USA) and Gene Tools (Syngene, USA) to analyze the bands.

### Statistical analysis

The data are presented in graphs showing average and standard deviation (SD). Unpaired Student-t test and ANOVA with on ranks tests were used to compare the data. The PCR results are presented as a ratio of the calibrator gene HPRT and presented in arbitrary units (AU). Differences were considered statistically significant with p less than 0.05. All statistical analyses were performed with the aid of GraphPad PRISM®.

## Results

### Malaria–associated acute kidney injury development during *P. berghei* ANKA infection

At day 14 after infection, parasitemia reached 65%, (Figure 1A), and none of *P. berghei*-infected mice became moribund, suggesting that they are resistant to development of cerebral malaria (Figure 1B). Next, we observed that serum creatinine and blood urea nitrogen (BUN) levels were markedly increased in infected BALB/c mice (Figure 1C and D). Additionally, we observed a decrease of total erythrocyte PpIX concentration in infected mice (Fig. 1E).

### *P.berghei* ANKA infection increase the proinflammatory profile during renal injury

The *P. berghei* ANKA infection significantly increases gene expression of IFN-γ on days 5 and 7 after infection (Fig. 2A). Analysis of mRNA expression of ICAM-1 showed a progressive increase in the response to the presence of the parasite, which reached significant values on 6 and 7 after infection (Fig. 2B). The mRNA expression of iNOS was also increased from day 3 after infection, although these differences were not statistically significant (Fig. 2C). These data are in agreement of increase of protein expression of IL-1β, IL-6 and TNF-α detected in renal tissue, induced by *P. berghei* ANKA (Fig. 2D–F). Corroborating those data, we observed that NF-κB pathway was activated in renal tissue from day 3 after infection by *P. berghei* ANKA (Fig. 2G).

### Pro-oxidant activity of oxidative stress during *P. berghei* ANKA malaria infection

The participation of the products generated by oxidative stress in the development of malaria-associated AKI was assessed by analysis of presence of heme and lipoprotein oxidation. We observed that plasma levels of toxic heme in BALB/c mice were significantly higher at days 7 and 12 after infection (Figure 3A). The conjugated dienes formation (Figure 3B) and plasma levels of oxidized LDL (Figure 3C) were significant up regulated during infection by *P. berghei* ANKA. In addition, we observed that *P. berghei* ANKA modulates a late expression of lectin-like oxidized LDL receptor-1 (LOX-1) mRNA expression in kidney tissue of BALB/c mice from day 5 after infection (Figure 3D).

### *P. berghei* ANKA infection compromise Renal Endothelial Permeability

Next, we investigated the changes in renal vascular endothelium, assessed by Evans Blue albumin dye protein leak [24]. We observed that infection with *P. berghei* ANKA induced a progressive increase of renal vascular permeability, as compared to non-infected controls (Figure 4A). Indeed, the histological examination of kidney sections showed that infection with *P. berghei* ANKA led to changes in renal architecture that ranges from a mild mononuclear cell infiltration at day 7 after infection to outstanding proinflammatory hypercellularity at day 15 after infection (Figure 4B). The morphological analysis of kidney sections showed changes that characterize acute tubule-interstitial nephritis (TIN). The bioplex analysis revealed a downregulation of vascular endothelial growth factor VEGF (Figure 5A) and an increase of KC, MCP-1 and MIP-1α point to neutrophils, macrophages and polymorphonuclear leukocytes as the most abundant leukocyte infiltration during malaria infection (Figure 5B, C and D).

### Sequestration and adhesion of infected red blood cells into the kidney

When kidney section was exposed under polarized light [25], we observed a marked deposition of malaria pigment hemozoin dispersed mostly at glomeruli and vascular endothelium (Figure 6A). Moreover, pigment was also observed at clusters of inflammatory infiltration (data not show). The granules of the pigment hemozoin were not observed at H&E standard microscopy (Figure 6A). The measurement of total brightly birefringent granules of the pigment was detected from day 3 after infection, the first time point assessed (Figure 6B). Quantitative approach by real time RT-PCR showed an upregulation of mRNA of *P. berghei* ANKA parasite at renal tissue from day 3 after infection confirmed the presence of parasite at renal tissue (Fig 6C). Further, using a set of *ex vivo* adherence assay, we verify that iRBC adhesion was increased on renal tissue sections from infected mice (7^th^ day pos infection) when compared with sections from the control group (Figure 7).

### Renal hypoxia and apoptosis during infection by *P. berghei* ANKA

In order to evaluate the extension of tissue damage during infection, we quantified renal tissue hypoxia and cellular apoptosis during malaria infection. Indeed, we observed a progressive increase of tissular hypoxia in kidney of infected mice (Figure 8A and B) and mRNA expression of HIF1-α (Figura 8C). Moreover, apoptosis was detected in renal tissue of infected mice when compare with non-infected control (Figure 8D).

### Heme detoxification and production of cytoprotective molecules during malaria-associated AKI

As oxidative stress mediated by toxic heme triggers an upregulation of cytoprotective and anti-inflammatory molecules, we determined the contribution of HO-1 as a mediator of the protection against malaria-associated AKI through modulating of anti-inflammatory response. We found a down-regulation of mRNA expression of HO-1 in renal tissue (Fig. 9A), as well as a decrease of plasma level of indirect bilirubin (Fig. 9B), a product of catabolism of toxic heme, TGF-β (Fig. 9C) and IL-10 (Fig. 9D) when compare with respective controls on day 0 after infection.

## Discussion

In the current study, we provide evidence that describe changes in the pathophysiology of kidney in an experimental model of severe malaria resembling to malaria-associated AKI in *P. falciparum* malaria. Impairment of renal function during malaria infection has been notified by clinical reports [26], [27] and it is an important life-threatening complication of malaria infection that goes beyond the classical clinical symptoms of *plasmodium*. The adversities to access of medical services, or delay in diagnosis in their place of origin, are implicated in the severity of disease [28]. The onset of kidney injury in BALB/c infected mice come out from day 4 after infection and the incidence of renal failure was confirm through manifestations such as increased of plasma creatinine and blood urea nitrogen (BUN) levels, as well as a decrease of total erythrocyte PpIX concentration in infected mice. This data reinforces the idea that the decrease of fluorescence emission of erythrocyte protoporphyrin IX, an intermediate product in the biosynthesis of heme, may be set up as a marker of several diseases as renal injury [19].

The pathophysiology of severe malaria are usually associated with a polyclonal activation of the immune system and comprehends a complex network with production of reactive oxygen and nitrogen species, exacerbated production of proinflammatory cytokines such as IFN-γ, TNF-α, IL-6, IL-1 and IL-8, as well by nuclear translocation of NF-κB [29]–[31]. In agreement with this notion, our study demonstrated that malaria infection markedly increase IFN-γ mRNA expression, as well protein expression of IL-1β, IL-6 and TNF-α in renal tissue of *P. berghei* ANKA infected mice, consistent with a previous study [6]. The up-regulation of protein expression of IKK in renal tissue support the idea that NF-kB pathway is required for this proinflammatory profile, as well as iNOS gene expression during malaria infection [32], [33].

Malaria-associated AKI is proposed to be a consequence of parasite adhesion as well as exacerbated immune response against products of oxidative stress released during infection [1]. The destruction of erythrocytes during blood stage of infection accumulates high levels of toxic free heme in circulation that, in turn, has the ability to induce oxidative stress from production of hydroxyl radicals via the Fenton/Haber-Weiss reaction [34]. Moreover, heme-derived oxidative stress is considered to be a main factor in the iron-induced lipid peroxidation resulting in the formation of oxidized LDL (ox-LDL) [8]. The results presented here strongly suggest that plasma oxidizability in BALB/c infected mice may results to free radicals generated from increased plasma levels of heme. Therefore, our data might add new insights to previous findings demonstrating the lipid peroxidation mediated by heme-induced oxidative stress during infection by *P. berghei* ANKA. Plasma oxidation assay measured by dienes absorption at 234 nm provide results as observed at ox-LDL plasma levels and it is a well-known index to determine oxidizability of plasma lipoproteins [22]. In addition, the mRNA expression of lectin-like oxidized LDL receptor (LOX-1) was also marked increased in renal tissue of infected mice. LOX-1 could be rapidly expressed in endothelial cells, macrophages, vascular smooth muscle cells and glomerular mesangial cells induced by products of oxidative stress, as well as pro-inflammatory cytokines [35]–[37]. The overproduction of ox-LDL triggers adverse effect in the progression of vascular lesions, generation of reactive oxygen species during infection and glomerulosclerosis [38]. The maintenance of pro-inflammatory state by ox-LDL plays an important role to modulate the up-regulation of ICAM-1, that induces adverse outcomes on renal microvascular permeability through leukocyte adherence, sequestration and adhesion of infected red blood cells (iRBC) to renal endothelial cells [24], [39], [40]. Addionally, ox-LDL also increases iNOS expression in renal tissue, during an intestinal ischemia/reperfusion injury [41]. iRBC sequestration at the microvascular site is an important feature of severe malaria. It has been shown that *P. falciparum* iRBC cytoadherence occurs via interactions of parasite surface antigen to endothelial receptor including ICAM-1 and CD36 [42], [43]. In this work we found an increase of ICAM-1 expression on renal tissue from *P. berghei* infected mice at day 7 post infection. Interestingly, *ex vivo* adhesion assays using sections from renal tissue from infected mice at this time-point show increased iRBC adhesion. Taken together, these results suggest that *P. berghei* interaction with the renal tissue can occur via ICAM-1. Therefore, we assume that exposition of endothelial cells to products of oxidative stress and parasite load plays a crucial role to endothelial activation and microvascular dysfunction in infected kidneys, concomitant with a markedly up-regulation of ICAM-1 in renal tissue. The cytoadherence of infected erythrocytes as well recruitment of monocytes, neutrophils and polymorphonuclear leukocytes, during pathogenesis of malaria-associated AKI could potentially contribute to renal hypoxia. In addition, an up-regulation of hypoxia inducible factor-1a (HIF-1α) mRNA and decrease of angiogenic factors protein expression (VEGF) in renal tissue can further induce morphological modifications. Changes in vascular permeability observed were quite expected, since microvascular dysfunction has been described before in the pathogenesis of ischemic-induced AKI. Recruitment of inflammatory cells during pathogenesis of malaria-associated AKI is in line with previous observations about involvement of infiltrating cells to increase vascular permeability [24]. This proinflammatory state also contributes to increase the occurrence of apoptotic events [44].

Usually, the exposition of host endothelial cells to free heme triggers an up-regulation of HO-1, an inducible enzyme that catalyzes the degradation of toxic heme [45]. In response to oxidative stress, HO-1 limits inflammation-associated tissue damage through the generation of product of catabolism of toxic heme as molecules of CO, bilirubin and ferritin[^8^]. Previous reports from our group have demonstrated a cytoprotective role of HO-1 in models of renal injury and ischemia and reperfusion events [20], [46], [47]. Moreover, HO-1 also prevents the development of experimental cerebral malaria (ECM), modulates the pro-inflammatory response during liver stage of *P. berghei* ANKA infection, as well as prevents hepatic injury in a noncerebral severe malaria infection [48]–[50]. Despite of above observations, we have found an impairment of mRNA expression of HO-1 in renal tissue, even as a decreased plasma level of indirect bilirubin. Anti-inflammatory and cytoprotective molecules were also down-regulated.

Taken together, our data suggest that both, proinflammatory molecules and products of oxidative stress have a central role to development of the pathogenesis of malaria-associated AKI. Our results also suggest that the loss of integrity of the renal vascular endothelium during infection are multifactorial in origin and may be related to increased toxic heme levels, reactive oxygen and nitrogen species, as well high levels of proinflammatory molecules. Modifications in the permeability of renal vascular endothelium, the final event of the combination of oxidative insult generated during infection, decreased O2 delivery to cells and tissues and contributed to increase hypoxic microenvironments. Moreover, the extent of ROS-induced oxidative damage can be exacerbated by decreased efficiency of antioxidant and cytoprotetive defense mechanisms.
